# MRI-derived cardiac washout is slowed in the left ventricle and associated with left ventricular non-compaction in young patients with cryptogenic ischemic stroke

**DOI:** 10.1007/s10554-022-02643-7

**Published:** 2022-05-30

**Authors:** Lauri Lehmonen, Jukka Putaala, Pauli Pöyhönen, Jouni Kuusisto, Jani Pirinen, Juha Sinisalo, Vesa Järvinen

**Affiliations:** 1 Radiology, HUS Diagnostic Center, Helsinki University Hospital and University of Helsinki, Haartmaninkatu 4, PO Box 340, 00029 Helsinki, Finland; 2 Neurology, Helsinki University Hospital and University of Helsinki, Helsinki, Finland; 3 Heart and Lung Center, Helsinki University Hospital and University of Helsinki, Helsinki, Finland; 4 Clinical Physiology and Nuclear Medicine, HUS Diagnostic Center, Helsinki University Hospital and University of Helsinki, Helsinki, Finland

**Keywords:** Magnetic resonance imaging, Cardiac, First-pass, Left atrial appendage, Left atrium, Stroke

## Abstract

To elucidate underlying disease mechanisms, we compared transition of gadolinium-based contrast agent bolus in cardiac chambers in magnetic resonance imaging between young patents with cryptogenic ischemic stroke and stroke-free controls. We included 30 patients aged 18–50 years with cryptogenic ischemic stroke from the prospective Searching for Explanations for Cryptogenic Stroke in the Young: Revealing the Etiology, Triggers and Outcome (NCT01934725) study and 30 age- and gender-matched stroke-free controls. Dynamic contrast-enhanced T1-weighted first-pass perfusion images were acquired at 1.5 T and analyzed for transit time variables, area under curves, relative blood flow, and maximum and minimum enhancement rates in left atrial appendage, left atrium, and left ventricle. These data were compared with previously published left ventricular non-compaction data of the same study population. Arrival time of contrast agent bolus in superior vena cava was similar in patients and controls (6.7[2.0] vs. 7.1[2.5] cardiac cycles, P = 0.626). Arrival and peak times showed comparable characteristics in patients and controls (P > 0.535). The minimum enhancement rate of the left ventricle was lower in patients than in controls (− 28 ± 11 vs. − 36 ± 13 1/(cardiac cycle), P = 0.012). Area under curves, relative blood flow, and other enhancement rates showed no significant differences between patients and controls (P > 0.107). Relative blood flow of cardiac chambers correlated with non-compacted left ventricular volume ratio (P < 0.011). Our results indicate slower washout of contrast agent and blood flow stagnation in the left ventricle of young patients with cryptogenic ischemic stroke. The washout was associated with left ventricular non-compaction, suggesting conditions favoring formation of intraventricular thrombosis.

## Introduction

Up to 50% of ischemic strokes in adults younger than 50 years are cryptogenic by nature, that is, without evident etiology based on routine diagnostic evaluation [1]. Cryptogenic ischemic strokes have been suggested to be frequently thromboembolic by mechanism, harboring proximal sources such as the heart [2]. We recently found that left atrial dynamics were altered based on echocardiographic findings [3], and that left ventricular non-compaction (LVNC) with normal ejection fraction was associated with cryptogenic ischemic stroke at young age independent of right-to-left shunt [4]. We hypothesized that prolonged circulation in different cardiac compartments assessed with cardiac magnetic resonance imaging (MRI)-based dynamic contrast enhancement (DCE) might provide further evidence of left-side cardiac pathology in young patients with cryptogenic ischemic stroke.

Contrast agent characteristics, such as transit times in different compartments of the body, have been commonly derived in diagnostic nuclear medicine using time-activity curves [5]. Analogously, MRI can be used to non-invasively produce time-intensity curves with dynamic MRI techniques, with precise anatomical location [6]. DCE MRI perfusion techniques have been applied in several cardiac disorders [7, 8], also in studies of the brain and oncological research [9–11]. With DCE perfusion, repeated T1-weighted images are obtained during contrast agent administration; DCE relies on the T1 shortening of gadolinium-based contrast agent, where a regional increase in the MR signal (shortening of T1 relaxation time) is caused by the gadolinium concentration, which depends on intravascular gadolinium and accumulation of gadolinium [12]. DCE perfusion images can be used to derive several semi-quantitative perfusion parameters pertaining to tissue perfusion and microvascular status [13]. DCE first-pass analysis has been employed in the circulation analysis of different compartments of the heart itself. Recently, MRI-derived first-pass analysis has been used to display differences in central circulation transit times (TTs) in patients with heart failure and preserved or reduced ejection fraction [14].

Here, we aimed to compare first-pass circulation characteristics of the cardiac chambers between young patients with cryptogenic ischemic stroke and stroke-free controls. Specifically, we sought to determine whether circulation was prolonged in the left atrial appendage (LAA), left atrium (LA), and left ventricle (LV), as well as to link first-pass characteristics to LVNC.

## Materials and methods

This is a substudy of the prospective multicenter Searching for Explanations for Cryptogenic Stroke in the Young: Revealing the Etiology, Triggers and Outcome (SECRETO; NCT01934725) study. SECRETO is an international prospective multicenter case–control study of young adults. In this substudy, we included 30 patients with cryptogenic ischemic stroke aged 18–50 years and 30 age- and gender-matched stroke-free controls from Helsinki University Hospital. The SECRETO study has been approved by the Ethics Committee of the Helsinki and Uusimaa Hospital District, and each participant provided written informed consent. Details of the study protocol have been published previously [15, 16]. Briefly, patients are enrolled in SECRETO after standardized minimum diagnostic procedures, including MRI of the brain, imaging of intracranial and extracranial vessels with computed tomography, angiography, or MRI, and cardiac imaging to rule out established causes of ischemic stroke. The cardiac work-up comprised step-by-step transthoracic echocardiography, transesophageal echocardiography with bubble test [16], transcranial Doppler ultrasound with bubble test, 12-lead electrocardiogram, and at least 24-h Holter electrocardiogram. Patients were excluded based on the following criteria: (1) Baseline minimum tests not obtained in the 1st week following stoke onset, including brain MRI and routine blood tests; (2) Other baseline minimum tests not obtained within the first 2 weeks following stroke onset, including imaging of cervicocephalic arteries, transesophageal or transthoracic echocardiography, 24-h Holter electrocardiogram, if patient was otherwise not eligible for the study, or if informed consent was not obtained from the patient or a proxy. Patients were age- and gender-matched to stroke-free controls in a 1:1 fashion. The stroke-free controls were searched in a random fashion through population registers. A selection of 20 potential controls per each patient were identified and invited by letter one by one, until a positive response to participate was obtained.

**Fig. 1:**
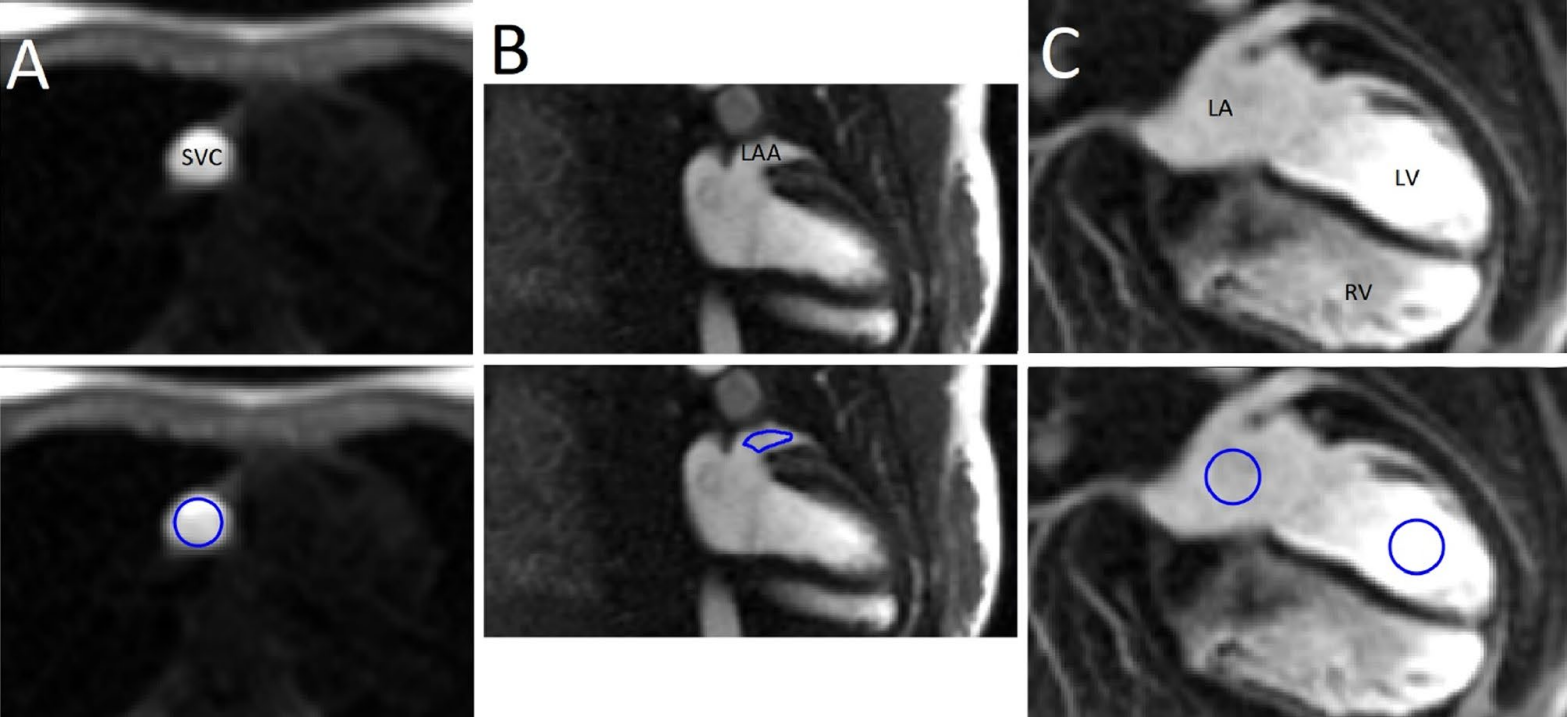
The four slice groups used to acquire first-pass circulation images, and example regions of interest used in the analysis. **a** Group 1, transverse view showing superior vena cava (SVC). **b** Groups 2 and 4, modified two-chamber view showing left atrial appendage (LAA). **c** Group 3, four-chamber view showing left atrium (LA), left ventricle (LV), and right ventricle (RV). Examples at time of peak signal intensity

All subjects in the substudy underwent cardiac MRI using a 1.5 T MAGNETOM Avanto^fit^ MR scanner (Siemens Healthcare, Erlangen, Germany). The main image acquisition protocol has been published previously [4]. The protocol included half-Fourier single-shot turbo spin echo sequence, to cover the entire heart in transversal view. This series was used to plan the balanced steady-state free precession cine sequences in transversal, two-chamber, three-chamber, four-chamber, and right-ventricular outflow-tract views. For the purposes of the present study, first-pass perfusion images were acquired simultaneously with gadolinium-based contrast agent (gadoterate meglumine, Dotarem, 0.2 mmol/kg at a rate of 4.5 ml/s) administration. The first-pass perfusion sequence was a prospective ECG-gated 2D motion-corrected saturation recovery steady-state free precession sequence consisting of four slice groups, each of which had an inversion time of 100 ms, field of view of 270 × 360 mm, repetition time of 180 ms, echo time of 1.2 ms, 12° flip angle, and 8 mm slice thickness. First-pass images were acquired in four slice groups to visualize the input: superior vena cava (SVC, transversal view; 1st group), LAA and LA (modified two-chamber view; 2nd and 4th groups), and LV (four-chamber view; 3rd group) (Fig. 1). The view for LAA was created using short-axis cine images to visualize the most proximal and distal parts of LAA. The LAA view was acquired twice per cardiac cycle to maximize temporal resolution. The sequence had 60 sample points for each slice group, and a total duration of around 60 s, depending on the participant’s heart rate. The location of the selected slice groups was verified for each participant before contrast agent administration with a dummy perfusion sequence of two sample points.

**Fig. 2:**
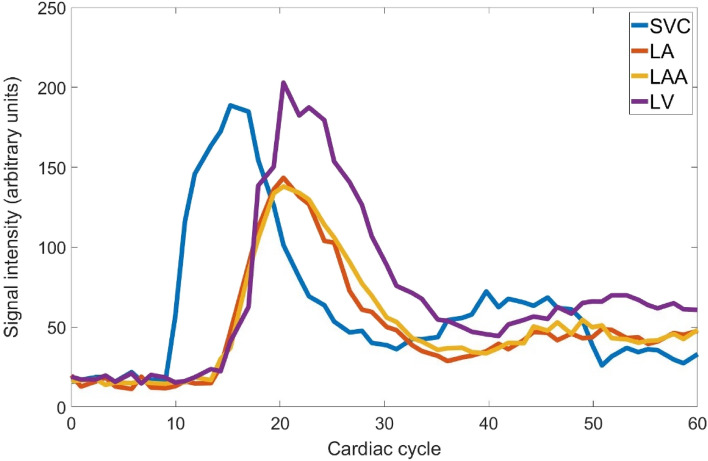
Example time-intensity curves of superior vena cava (SVC), left atrium (LA), left atrial appendage (LAA), and left ventricle (LV) without baseline correction

Contrast agent administration was performed in two phases to shorten the initial bolus duration and to visualize the washout (in addition to the wash in) of contrast agent. The bolus injection was started at the same time as the perfusion sequence, with 1/2 of the bolus injected. The final 1/2 of the bolus was injected after the perfusion sequence was acquired. Our experience with one-phase bolus injections has shown that no clear washout of the contrast agent occurs during first pass, leaving the MRI signal intensity at a high level compared with baseline; this is suboptimal for semi-quantitative circulation analysis of the washout phase, and the calculation of such parameters as full width at half maximum (FWHM) or full width at tenth maximum (FWTM) is not possible. The use of two-phase bolus injection provided a clear washout phase. A second signal increase in the time-intensity curves is caused by recirculation, and only the first peak in the curves was used for analysis (Fig. 2).

The first-pass circulation analysis was performed with Segment software v3.2 R8757 (Medviso AB, Lund, Sweden) by an experienced medical physicist (LL) blinded to patient-control status of each participant. Time (cardiac cycles)-intensity (arbitrary units) curves for SVC, LA, and LV were obtained by placing circular regions of interest (ROIs) of at least 150 mm^2^ in the transverse and four-chamber images. LAA was analyzed by drawing a custom size and shape ROI for each subject, to match the visible part of LAA in each time frame in the modified left ventricular two-chamber images, as the shape and size of the LAA differed between study participants. Mean signal intensity as function of time was extracted for each ROI (Fig. 2). As the signal intensity of the image sequence used was not zero at the beginning of the sequence, the mean signal intensity inside the analysis ROI of the first three datapoints was used to correct for the baseline of each time-intensity curve.

**Fig. 3:**
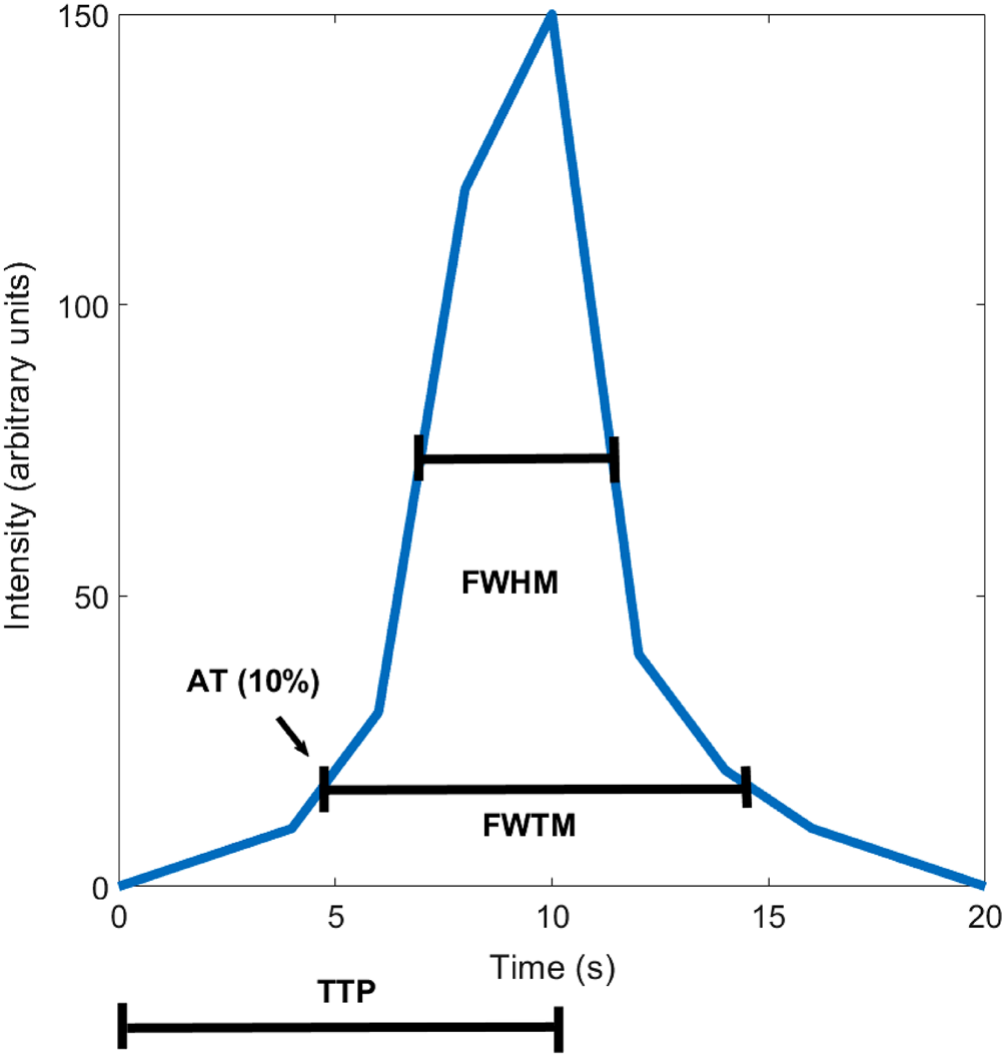
A one-peak example curve denoting the definition of arrival time (AT), time-to-peak (TTP), full width at half maximum (FWHM), and full width at tenth maximum (FWTM)

The time-intensity curves were used to calculate several semi-quantitative circulation parameters: arrival time (AT, time at 10% of maximum intensity), time-to-peak (TTP, measured from the start of the sequence), FWHM, and FWTM for SVC, LAA, LA, and LV. An example of the analyzed time parameters is given in Fig. 3. Additionally, TTs, defined as the time between the peaks of the time-intensity curves, were calculated from SVC to LAA, SVC to LA, SVC to LV, and LA to LAA. All time variables were normalized to subject RR-interval, and results were gathered per cardiac cycle (cc). The time-intensity curve analysis was performed with MATLAB R2019A (TheMathWorks, Inc., Natick, MA, USA). Using the time-intensity curve data, we calculated the maximum and minimum enhancement rates (peak time derivatives), area under curves (AUCs) with trapezoidal numerical integration using the length of FWTM as integration range, and relative blood flow (rBF) defined as AUC/FWTM for SVC, LAA, LA, and LV.

Finally, we sought correlations between the circulation parameters of this study and previously published LVNC related data in the same study population [4]. The LVNC data comprised absolute and indexed left ventricular non-compacted mass (NC LVM and NC LVMI), compacted mass (C LVM and C LVMI), non-compacted to compacted mass ratio (NC to C LVM), and non-compacted left ventricular volume ratio (% of end-diastolic volume, NC to LVEDV). The LVNC data were calculated using cardiac cine images.

Statistical analysis was performed with IBM SPSS Statistics for Windows, version 27 (IBM Corp., Armonk, NY, USA). Normality of the analyzed continuous variables was assessed using the Kolmogorov–Smirnov test. Continuous variables are presented as either mean ± standard deviation or median (interquartile range), depending on data normality. Differences between patients and controls were evaluated using Student’s *t*-test if data were normally distributed or otherwise with Mann–Whitney U test. Correlations were calculated using Pearson correlation coefficient if data were normally distributed or otherwise using Spearman’s rank correlation coefficient. P-values < 0.05 were considered significant.

## Results

Demographic data, LV volumetric data, and LVNC data of the study population are presented in Table 1. No significant differences in chamber volumes or ejection fractions emerged between patients with cryptogenic ischemic stroke and stroke-free controls. The previously published non-compacted LV mass, non-compacted to compacted mass ratio, and non-compacted LV volume ratio were all significantly different between patients and controls (P < 0.045).

**Table 1:** Demographic data, left ventricular volumetric data, and non-compaction data of the study population

Parameter	Patients (N = 30)	Controls (N = 30)	P value
Age (years)	41 ± 8	41 ± 8	0.564
Number of females, N (%)	15 (50)	15 (50)	1
BMI (kg/m^2^)	28.4 ± 5.0	26.9 ± 5.2	0.875
BSA (m^2^)	2.04 ± 0.24	1.93 ± 0.24	0.075
LAVImax (ml/m^2^)	44.5 ± 7.2	45.1 ± 10.1	0.790
LVEDVI (ml/m^2^)	84.5 ± 10.6	86.1 ± 15.3	0.646
LVESVI (ml/m^2^)	30.5 ± 6.1	31.0 ± 7.2	0.752
LVEF (%)	64.1 ± 4.7	63.8 ± 5.1	0.816
NC LVM (g)	30.0 ± 7.4	26.4 ± 10.1	0.014*
NC LVMI (g/m^2^)	14.0 (5.4)	12.7 (6.2)	0.045*
C LVM (g)	116.1 (40.1)	110.0 (34.3)	0.229
C LVMI (g/m^2^)	55.3 (14.5)	57.1 (10.9)	0.797
NC to C LVM (%)	25.6 ± 4.2	22.8 ± 6.0	0.015*
NC to LVEDV (% of EDV)	17.6 ± 2.9	15.7 ± 3.8	0.004*

Data are presented as mean ± standard deviation or as median (interquartile range)

*BMI* body mass index, *BSA* body surface area, *LAVImax* indexed left atrial maximum volume, *LVEDVI* indexed left ventricular end-diastolic volume, *LVESVI* indexed left ventricular end-systolic volume, *LVEF* left ventricular ejection fraction, *NC LVM* non-compacted left ventricular mass, *NC LVMI* indexed non-compacted left ventricular mass, *C LVM* compacted left ventricular mass, *C LVMI* indexed compacted left ventricular mass, *NC to C LVM* non-compacted to compacted left ventricular mass, *NC to LVEDV* non-compacted to left ventricular end-diastolic volume

*Significant difference (P < 0.05)

Results for the time variables in the first-pass circulation analysis are displayed in Table 2. The contrast agent AT in SVC was similar in patients and controls (P = 0.626). AT, TTP, FWHM, and FWTM of LAA, LA, SVC, and LV showed no significant differences between patients and controls (P > 0.133). TTs between different cardiac compartments were also similar in patients and controls (P > 0.605). The closest to significant differences were found in FWTM of LAA and LA, (21.6 ± 5.2 vs. 20.8 ± 4.2 cardiac cycles, P = 0.133, and 22.5 ± 7.1 vs. 20.7 ± 3.3 cardiac cycles, P = 0.207; respectively).

**Table 2:** Results for the time variables of the first-pass circulation analysis in patients with cryptogenic ischemic stroke (N = 30) and stroke-free controls (N = 30)

Parameter	Patients (N = 30)	Controls (N = 30)	P value
Heart rate (bpm)	66 ± 11	70 ± 13	0.206
AT SVC (cc)	6.7 (2.0)	7.1 (2.5)	0.626
TTP SVC (cc)	11.9 (5.0)	12.5 (4.7)	0.988
FWHM SVC (cc)	6.2 (3.0)	7.3 (2.1)	0.337
FWTM SVC (cc)	14.9 ± 3.2	15.7 ± 3.8	0.778
AT LAA (cc)	12.4 (2.3)	13.5 (3.6)	0.657
TTP LAA (cc)	19.6 (5.1)	19.8 (6.9)	0.929
FWHM LAA (cc)	11.1 (5.1)	10.4 (3.2)	0.733
FWTM LAA (cc)	22.5 ± 7.1	20.7 ± 3.3	0.207
AT LA (cc)	12.2 (2.8)	13.0 (3.6)	0.535
TTP LA (cc)	18.7 (5.5)	18.1 (5.2)	0.790
FWHM LA (cc)	10.5 (5.6)	10.4 (2.7)	0.446
FWTM LA (cc)	21.6 ± 5.2	19.7 ± 4.3	0.133
AT LV (cc)	13.3 (4.0)	13.7 (4.0)	0.657
TTP LV (cc)	21.5 (8.0)	20.1 (6.8)	0.544
FWHM LV (cc)	11.4 (6.0)	10.8 (2.5)	0.758
FWTM LV (cc)	21.7 ± 4.8	20.8 ± 4.2	0.429
TT SVC to LAA (cc)	6.9 (3.1)	7.0 (3.0)	0.871
TT SVC to LA (cc)	6.2 (2.3)	6.6 (2.7)	0.929
TT SVC to LV (cc)	8.1 (3.4)	8.2 (2.9)	0.605
TT LA to LAA (cc)	1.0 (1.1)	0.9 (1.3)	0.947

Results are presented as mean ± standard deviation or as median (interquartile range)

*bpm* beats per minute, *AT* arrival time, *SVC* superior vena cava, *cc* cardiac cycle, *TTP* time-to-peak, *FWHM* full width at half maximum, *FWTM* full width at tenth maximum (for more detailed information, see Fig. 3), *LAA* left atrial appendage, *LA* left atrium, *LV* left ventricle, *TT* transit time

The calculated AUCs, rBFs, and enhancement rates showed mostly similarities between patients and controls (Table 3). However, the minimum enhancement rate (Dmin) of the LV was significantly lower in patients than in controls (− 28 ± 11 vs. − 36 ± 13 1/cc, P = 0.012), indicating slower washout of contrast agent from the LV.

**Table 3:** Results for area under curves, relative blood flow, and maximum and minimum enhancement rates of first-pass circulation in patients with cryptogenic ischemic stroke (N = 30) and stroke-free controls (N = 30)

Parameter	Patients (N = 30)	Controls (N = 30)	P value
AUC SVC (cc)	1326 (1463)	1588 (1010)	0.317
rBF SVC	97 ± 36	101 ± 34	0.651
Dmax SVC (1/cc)	92 ± 42	88 ± 37	0.667
Dmin SVC (1/cc)	− 46 (40)	− 43 (28)	0.514
AUC LAA (cc)	2440 (2196)	2217 (1242)	0.367
rBF LAA	117 ± 42	111 ± 44	0.715
Dmax LAA (1/cc)	53 (26)	49 (23)	0.469
Dmin LAA (1/cc)	− 19 (12)	− 22 (10)	0.391
AUC LA (cc)	2382 (1618)	1814 (1070)	0.179
rBF LA	111 ± 32	106 ± 34	0.813
Dmax LA (1/cc)	56 ± 19	60 ± 27	0.514
Dmin LA (1/cc)	− 19 (12)	− 22 (14)	0.143
AUC LV (cc)	2995 (3284)	3128 (2201)	0.871
rBF LV	176 ± 68	173 ± 63	0.972
Dmax LV (1/cc)	74 ± 32	88 ± 34	0.107
Dmin LV (1/cc)	− 28 ± 11	− 36 ± 13	0.012*

Results are presented as mean ± standard deviation or median (interquartile range)

*AUC* area under curve, *SVC* superior vena cava, *cc* cardiac cycle, *rBF* relative blood flow, *Dmax* maximum derivative, *Dmin* minimum derivative, *LAA* left atrial appendage, *LA* left atrium, *LV* left ventricle

*Significant difference (P < 0.05)

Significant correlations emerged between the relative blood flow of several cardiac chambers, and maximum and minimum enhancement rate of LA and LV, and LVNC variables in the entire study population (N = 60) (Table 4). The non-compacted left ventricular volume ratio correlated with the relative blood flow of LAA, LA, and LV (P < 0.011). The absolute non-compacted left-ventricular mass was also significantly correlated with both the maximum and minimum enhancement rates of LAA, and LA (P < 0.019).

**Table 4:** Correlations between the circulation parameters of this study and left ventricular non-compaction parameters (N = 60)

Parameter	NC LVM (g)		NC to C LVM (%)		NC to LVEDV (% of EDV)	
	R	P value	R	P value	R	P value
rBF LAA	− 0.32	0.013*	− 0.25	0.053	− 0.33	0.011*
rBF LA	− 0.37	0.004*	− 0.29	0.026*	− 0.36	0.005*
rBF LV	− 0.23	0.072	− 0.32	0.014*	− 0.34	0.009*
Dmax LAA (1/cc)	− 0.36	0.004*	− 0.22	0.096	− 0.35	0.006*
Dmin LAA (1/cc)	0.30	0.019*	0.03	0.783	0.24	0.066
Dmax LA (1/cc)	− 0.35	0.006*	− 0.40	0.001*	− 0.24	0.070
Dmin LA (1/cc)	0.37	0.004*	0.04	0.720	0.29	0.027*
Dmax LV (1/cc)	− 0.15	0.258	− 0.16	0.215	− 0.23	0.075
Dmin LV (1/cc)	0.24	0.063	0.20	0.136	0.36	0.005*

*NC LVM* non-compacted left ventricular mass, *NC to C LVM* non-compacted to compacted left ventricular mass, *NC to LVEDV* non-compacted to left ventricular end-diastolic volume (% of left ventricular end-diastolic volume), *rBF* relative blood flow, *LAA* left atrial appendage, *LA* left atrium, *LV* left ventricle, *Dmax* maximum derivative, *Dmin* minimum derivative, *R* Pearson correlation coefficient in case of normally distributed variables, and Spearman’s rank correlation coefficient otherwise

*Significant correlation (P < 0.05)

## Discussion

We utilized MRI-derived gadolinium first-pass analysis to study circulation in the hearts of young patients with cryptogenic ischemic stroke and stroke-free controls. We were able to detect a significant difference in the contrast agent washout in the left ventricles of our study population. The washout, together with relative blood flow, was significantly associated with the left ventricular non-compacted LV volume ratio, linking prolonged circulation to LVNC in cryptogenic ischemic stroke.

We used a two-phase contrast agent bolus injection to visualize contrast agent washout in addition to wash in and sampled the LAA twice per cardiac cycle to increase the temporal resolution. We still could not explicitly prove our first hypothesis that circulation is prolonged in LAA or LA in our patients with cryptogenic ischemic stroke relative to stroke-free controls. However, there was a significant difference in the minimum enhancement rate of the LV, which describes contrast agent washout. The differences in AT, TTP, FWHM, FWTM, AUC, and rBF in the LV of patients and controls were all non-significant, indicating similar circulation behavior. None of these parameters directly depict the rate of contrast agent wash in and washout, however. Importantly, patients and controls had no significant differences in left ventricular end-systolic or end-diastolic volumes, ejection fractions, or valvular insufficiencies that could explain the slower washout of contrast from the LV. Therefore, the observed difference in contrast agent washout is probably a true sign on LVNC being the cause of blood flow stagnation. Whether the patients with slight LVNC finding but not fulfilling the criteria of non-compaction cardiomyopathy have actual myocardial pathology, or merely a normal variation with increased risk of thrombosis, cannot be concluded from this imaging-only study.

To further assess possible pathology explaining the difference in contrast washout between patients and controls, we assessed its correlation with previously found LVNC in the study population [4]. In the previous study, Pöyhönen et al. showcased significantly higher non-compacted LV mass, ratio of non-compacted to compacted LV mass, and a higher non-compacted LV volume ratio. In the present study, we linked these findings by Pöyhönen et al. to several first-pass circulation measures. Indeed, we found multiple circulation parameters to be strongly correlated with LVNC parameters. The relative blood flow of LAA, LA, and LV all showed a significant correlation with non-compacted LV volume. Similarly, ratio of non-compacted volume to LV volume correlated with the wash in rate of LAA, while with LA and LV the correlation was significant in the washout phase. Taken together, the increasing ratio of non-compacted volume to LV end-diastolic volume was associated with the magnitude of circulation in LAA, LA, and LV. These findings further strengthen the association between LVNC and cryptogenic ischemic stroke in the young.

Since none of our patients fulfilled the criteria on non-compaction cardiomyopathy, the higher tendency for LVNC among the patients, compared to controls, merely reflects the continuous nature of risk. LVNC is a probably a risk factor for thromboembolism due to a larger area of endocardium, as well as increased amount of myocardial indentations and crypts, and in addition to this, we also found that LVNC causes stagnation of blood flow, hence making another contribution to the formation of thrombosis. However, due to the nature of our study, we cannot draw conclusions on whether any differences in secondary prevention can be of use in cryptogenic stroke patients with LVNC not fulfilling the criteria of non-compaction cardiomyopathy. However, the finding can be of use in further studies, and there might be a degree of LVNC below the limit of non-compaction cardiomyopathy criteria, causing a significant risk of intracardiac thrombosis.

The main strengths of our study are: All patients underwent a standardized and thorough clinical diagnostic work-up before they were labeled as having cryptogenic ischemic stroke. Our cardiac MRI protocol was rigorous to enable detection of subtle differences between patients and controls. The study also has limitations. Our sample size is relatively small, reflecting the high demands placed on all study participants. The MRI protocol is limited to the semi-quantitative analysis of the time-intensity circulation curves. No information on the native pre-contrast T1 values was available, which is a requirement for the quantitative contrast agent concentration calculation [6, 17]. Due to the non-linear relationship between DCE signal intensity and the T1 shortening caused by gadolinium, the absolute signal intensity values were not comparable between study subjects. Finally, due to the nature of case–control studies, some selection of participants is possible, and we can only demonstrate associations, not causality.

Contrast-enhanced cardiac MRI can be used to study circulation in different cardiac chambers. We observed a tendency for stagnation of blood flow in the left ventricle of young patients with cryptogenic ischemic stroke, with a link to LVNC pathology, suggesting conditions favoring formation of intraventricular thrombosis.
